# Pre-transplant gene profiling characterization by next-generation DNA sequencing might predict relapse occurrence after hematopoietic stem cell transplantation in patients affected by AML

**DOI:** 10.3389/fonc.2022.939819

**Published:** 2022-12-07

**Authors:** Elisabetta Metafuni, Viviana Amato, Sabrina Giammarco, Silvia Bellesi, Monica Rossi, Gessica Minnella, Filippo Frioni, Maria Assunta Limongiello, Livio Pagano, Andrea Bacigalupo, Simona Sica, Patrizia Chiusolo

**Affiliations:** ^1^ Dipartimento di Diagnostica per Immagini, Radioterapia Oncologica ed Ematologia, Fondazione Policlinico Universitario “A. Gemelli” Istituto di Ricovero e Cura a Carattere Scientifico (IRCCS), Rome, Italy; ^2^ Division of Haemato-Oncology, IEO European Institute of Oncology Istituto di Ricovero e Cura a Carattere Scientifico (IRCCS), Milan, Italy; ^3^ Sezione di Ematologia, Dipartimento di Scienze Radiologiche ed Ematologiche, Università Cattolica del Sacro Cuore, Rome, Italy

**Keywords:** AML, stem cell transplant (SCT), NGS, relapse, genes

## Abstract

**Background:**

In the last decade, many steps forward have been made in acute myeloid leukemia prognostic stratification, adding next-generation sequencing techniques to the conventional molecular assays. This resulted in the revision of the current risk classification and the introduction of new target therapies.

**Aims and methods:**

We wanted to evaluate the prognostic impact of acute myeloid leukemia (AML) mutational pattern on relapse occurrence and survival after allogeneic stem cell transplantation. A specific next-generation sequencing (NGS) panel containing 26 genes was designed for the study. Ninety-six patients studied with NGS at diagnosis were included and retrospectively studied for post-transplant outcomes.

**Results:**

Only eight patients did not show any mutations. Multivariate Cox regression revealed FLT3 (HR, 3.36; p=0.02), NRAS (HR, 4.78; p=0.01), TP53 (HR, 4.34; p=0.03), and WT1 (HR 5.97; p=0.005) mutations as predictive variables for relapse occurrence after transplantation. Other independent variables for relapse recurrence were donor age (HR, 0.97; p=0.04), the presence of an adverse cytogenetic risk at diagnosis (HR, 3.03; p=0.04), and the obtainment of complete remission of the disease before transplantation (HR, 0.23; p=0.001). Overall survival appeared to be affected only by grade 2–4 acute GvHD occurrence (HR, 2.29; p=0.05) and relapse occurrence (HR, 4.33; p=0.0001) in multivariate analysis.

**Conclusions:**

The small number of patients and the retrospective design of the study might affect the resonance of our data. Although results on TP53, FLT3, and WT1 were comparable to previous reports, the interesting data on NRAS deserve attention.

## Introduction

The term acute myeloid leukemia (AML) includes a heterogeneous group of hematological diseases described by the World Health Organization (WHO) in 2016 (1). AML is the most common acute leukemia in adults, with an overall incidence of approximately 2.5–3 cases per 100,000 people per year (2), which progressively increases with age, from 1.3 per 100,000 subjects under 65 years to 12.2 cases per 100,000 in those subjects over 65 years (3). Molecular studies have led to the identification of several chromosomal abnormalities and genetic mutations involving sequences encoding for genes responsible for hematological disease development. Some of these mutations, particularly those affecting epigenetic proteins DNA methyltransferase 3 alpha (DNMT3A) and Tet methylcytosine dioxygenase 2 (TET2), are weakly expressed in many individuals over 50 years. In 10%–40% of cases, the clone progresses into clonal hematopoiesis of undetermined potential, and a minority of them develop hematological neoplasms (age-related clonal hemopoiesis) (4). Among the genes reported as significantly associated with AML induction, there are genes involved in DNA methylation like isocitrate dehydrogenase (IDH1 and IDH2), DNMT3A, TET2, oncosuppressor genes like TP53, and spliceosome genes (5, 6). Karyotype abnormalities stratification according to European Leukemia Net predicts response and overall survival in patients with AML and guides the physicians in therapeutic choices (7). Approximately 60% of the newly diagnosed AML present gene mutations involving activation pathways with a relevant pathogenic role (8). The translocations t(8;21), t(15;17), and inv (16) define a favorable prognosis (9, 10), while complex karyotype, chromosome 5 or 7 monosomy, t(6;9), inv (3), and other mutations involving chromosome 11q predict poor outcome and treatment resistance (11). Gene mutational status results are likewise relevant in the large slice of individuals with normal karyotypes. In these patients, biallelic mutation of CCAAT enhancer binding protein alpha (CEBPA) and nucleophosmin 1 (NPM1) mutation, in the absence of Fms-related receptor tyrosine kinase 3 (FLT3) mutation, confer a favorable prognosis (9, 12, 13). On the other hand, FLT3-ITD mutation determines the poor outcome (14), especially when a high mutated/wild-type ratio is detected (15, 16). Mutated TP53, frequently associated with complex karyotype, is reported as an adverse prognostic factor (17) even when it represents the only mutation detected (18). Mutated DNMT3A predicts unfavorable outcomes (19), particularly when combined with mutated NPM1 and FLT3 (20). Partial tandem duplications of lysine methyltransferase 2A (KMT2A) have also been associated with a worse prognosis in normal karyotype AML (17, 18, 21). TET2 mutation described in the AML inactivates the enzyme and seems to confer a worse prognosis to the disease (21). The prognostic impact of IDH-1/IDH-2 mutations is less well established and is likely modified by concomitant mutations. In patients with normal karyotype, FLT3-ITD-negative, and NPM1 mutations, the IDH-1/IDH-2 mutation appears to improve overall survival (21). In 2017, the European Leukemia Net (ELN) published the classification of AML, according to molecular and cytogenetic risk, into favorable, intermediate, and adverse risk. Acute myeloid leukemia represents the major indication for allogeneic stem cell transplantation (HSCT), especially for patients in the adverse risk group and for those at high risk of relapse who had achieved a complete remission (7). Here, we wanted to evaluate how molecular mutations might affect the outcome of patients with acute myeloid leukemia submitted to allogeneic stem cell transplantation.

## Materials and methods

The study was conducted according to the Declaration of Helsinki and was approved by the ethics committee of the Fondazione Policlinico Universitario Agostino Gemelli IRCCS in Rome (Protocol ID 4417).

### Sample processing, DNA sequencing, and mutation analysis

We analyzed bone marrow mononuclear cells collected at the time of AML diagnosis. DNA was isolated using the Qiamp DNA Blood Mini Kit (Qiagen, Germantown, MD). A custom enrichment panel (Illumina, San Diego, CA) with target regions of 26 genes was designed using Illumina Design StudioTM software. Genes were selected based on the available evidence in myeloid neoplasms (**Table 1**). The custom panel consisted of 263 selected targets, 86,715 bp in cumulative target length, for a total of 2.088 probes. Unique dual-indexed paired-end libraries were created from high-quality double-stranded genomic DNA (gDNA) inputs of 10–1,000 ng, using the Nextera DNA Flex for Enrichment workflow, updated during the study with Illumina DNA Prep with Enrichment workflow. Libraries were sequenced on the MiniSeq sequencing system using the MiniSeq Mid Output kit (300 cycles) and setting up a paired-end run with 149 cycles per reading (2×149) and 10 cycles per index read. The resulting average depth of sequencing coverage was 1,000×. Sequence reads were initially aligned to the human genome (GRCh37/hg19) using the Burrows–Wheeler aligner. All enrichment values were calculated without padding (sequence immediately upstream and downstream). If any targeted region overlapped another region, the region positions have been adjusted to remove overlaps. For alignment, somatic variant caller (version 3.5.2.3) was selected. The variants were identified by BaseSpace Variant Interpreter Software Illumina. Functionally annotated variants were filtered accordingly to the following criteria: synonymous variants and variants located outside protein-coding regions were filtered; polymorphisms described in dbSNP (version 155) with a population frequency >1% were removed; and variants with coverage <30× and <10 supporting reads and variants with an allelic fraction (VAF) lower than 5% were filtered. The remaining variants, evaluated as candidate somatic mutations, were finally tagged as oncogenic using different criteria based on information retrieved from the literature, sequence conservation, and *in silico* prediction effect (22–25). NPM1 and FLT3 mutations were detected as previously described (26, 27). Patients reported as FLT3 positive were those with a high allelic ratio.

**Table 1:** NGS gene panel used.

GENE (exon)	ID Trascript	Mutation frequencyn. (%)
**ASXL1** (**13**)	NM_015338.5	13 (13.5)
**CALR** (**9**)	NM_004343.3	0 (0)
**CBL** (**8, 9**)	NM_005188.3	3 (3.1)
**CBLB** (**10**)	NM_170662.3	0 (0)
**CEBPA (all)**	NM_004364.4	10 (10.4)
**KIT** (**2, 8–11, 13, 17**)	NM_000222.2	4 (4.2)
**CSF3R** (**14–17**)	NM_156039.3	0 (0)
**CUX1 (all)**	NM_181552.3	0 (0)
**DNMT3A (all)**	NM_175629.2	29 (30.2)
**EZH2 (all)**	NM_004456.4	2 (2.1)
**IDH1** (**4**)	NM_005896.3	8 (8.3)
**IDH2** (**4**)	NM_002168.3	12 (12.5)
**IKZF1 (all)**	NM_006060.5	0 (0)
**JAK2** (**14**)	NM_004972.3	2 (2.1)
**KRAS**	NM_033360.3	1 (1)
**MPL**	NM_005373.2	0 (0)
**NRAS**	NM_002524	7 (7.3)
**RUNX1 (all)**	NM_001754.4	11 (11.5)
**SETBP1** (**4**)	NM_015559.2	0 (0)
**SF3B1** (**13–16**)	NM_012433.3	3 (3.1)
**SRSF2** (**1**)	NM_001195427	4 (4.2)
**TET2** (**3–11**)	NM_001127208.2	34 (35.4)
**TP53** (**2–11**)	NM_000546.5	6 (6.3)
**U2AF1** (**2–6**)	NM_006758.2	4 (4.2)
**WT1** (**7–9**)	NM_024426.4	7 (7.3)
**ZRSR2 (all)**	NM_005089.3	2 (2.1)

### Patients

We enrolled 96 patients with AML candidates to receive HSCT between 2016 and 2021 for which an AML NGS panel was available at diagnosis. Patient characteristics, data on AML diagnosis, features, previous treatment, and transplant conditions are reported in **Table 2**.

**Table 2:** Patients, disease, and transplant characteristics.

Total cohort	96 patients
*Sex M/F*	57 (70%)/39 (30%)
*Age at transplant*	56 years (17–73)
*Cytogenetic risk* (28) Favorable Intermediate Adverse	4 (4%) 79 (82%) 13 (14%)
*ELN risk* (7) Favorable Intermediate Adverse	15 (16%) 43 (45%) 38 (39%)
*Line of treatment before HSCT n.* None 1 2 3	4 (4%) 69 (72%) 19 (20%) 4 (4%)
*Median time to transplant*	183.5 days (30 to 645)
*HCT-CI* 0 1 2 3 4 ≥5	9 (9%) 11 (11%) 17 (18%) 18 (19%) 23 (24%) 18 (19%)
*Year of transplant* 2016–2018 2019–2021	29 (30%) 67 (70%)
*Disease status at transplant* Never treated CR1 CR2 PR Relapse/refractory	6 (6%) 54 (56%) 12 (13%) 6 (6%) 18 (19%)
*Conditioning* ABL RIC/NMA	42 (44%) 54 (56%)
*Donor* HLA Id Sibling Haploidentical Mismatched unrelated Matched unrelated	21 (22%) 32 (33%) 15 (16%) 28 (29%)
*Stem cell source* PB CB BM	67 (70%) 4 (4%) 25 (26%)
*GvHD prophylaxis* CSA+Cy CSA+MFA+Cy CSA+MFA CSA+MTX	4 (4%) 70 (73%) 2 (2%) 20 (21%)
*ATG*	18 (19%)

M, male; F, female; HCT-CI, hematopoietic cell transplant comorbidity index; CR, complete remission; PR, partial response; ABL, myeloablative; RIC, reduced intensity conditioning; NMA, non-myeloablative conditioning; PB, peripheral blood; CB, cord blood; BM, bone marrow; CSA, cyclosporine A; MFA, micofenolic acid; MTX, methotrexate; CY, cyclophosphamide post-transplant; ATG, anti-thymocyte globulins.

### Statistical analysis

Statistical analysis was realized using NCSS10 software. For each NGS mutation, the frequency was reported as an absolute value and as a percentage of the entire population. A comparison between continuous numerical variables among different groups was made using the Mann–Whitney U test. To identify the association between categorical variables, chi-square and Fisher’s exact test were used. One-year survival curves for overall survival (OS) were built with the Kaplan–Meier method, and a comparison between curves was assessed with the log-rank test. Cumulative incidence of relapse was calculated considering death as a competitive event, and a comparison between curves was made using Gray’s test. Cumulative incidence of transplant-related mortality (TRM) was calculated considering relapse occurrence and death by other causes as competitive events. The Cox regression method was used to identify variables affecting the time-dependent outcomes OS, TRM, and DFS. Variables with a p-value <0.1 in univariate analysis were included in multivariate one. Statistical significance was assigned for p-value <0.05.

## Results

Only eight patients (8.3%) did not show any mutation. Twenty-three patients (24%) had one mutation, 25 patients (26%) had two mutations, the other 25 patients (26%) had three mutations, 10 patients (10.5%) had four mutations, and 5 patients (5.2%) had five mutations. Mutation frequencies are listed in **Table 1**.

### Mutations and leukemia features

Total white blood count at AML diagnosis was higher in patients with FLT3 mutation (64.7 *vs*. 7.8×10^9^/L, p=0.0004), NPM1 mutation (35.9 *vs*. 6.2×10^9^/L, p<0.0001), and DNMT3A mutation (29.5 *vs*. 7.7×10^9^/L, p=0.001), whereas it was lower in patients with IDH2 mutation (2.9 *vs*. 10.9×10^9^/L, p=0.04) and RUNX1 mutation (3.0 *vs*. 12.4×10^9^/L, p=0.02). In relation to bone marrow blasts percentage at AML diagnosis, it was higher in patients with FLT3 mutation (74% *vs*. 45%, p=0.007) and NPM1 mutation (70% *vs*. 41%, p=0.002). Furthermore, hemoglobin levels at AML diagnosis were higher in patients with ASXL1 mutation (10.5 *vs*. 8.9 g/dl, p=0.006), whereas platelet count was higher in patients with DNMT3A mutation (77 *vs*. 47×10^9^/L, p=0.02). Finally, we observed that FLT3 mutation was frequently combined with NPM1 mutation (83%, p<0.0001) and DNMT3A mutation (67%, p=0.0001). Similarly to that, NPM1 mutation was frequently revealed together with FLT3 mutation (47%, p<0.0001) and DNMT3A mutation (59%, p<0.0001), and DMT3A mutation was frequently present together with FLT3 mutation (41%, p<0.0001) and NPM1 mutation (65%, p<0.0001).

### Relapse

**Figure 1:**
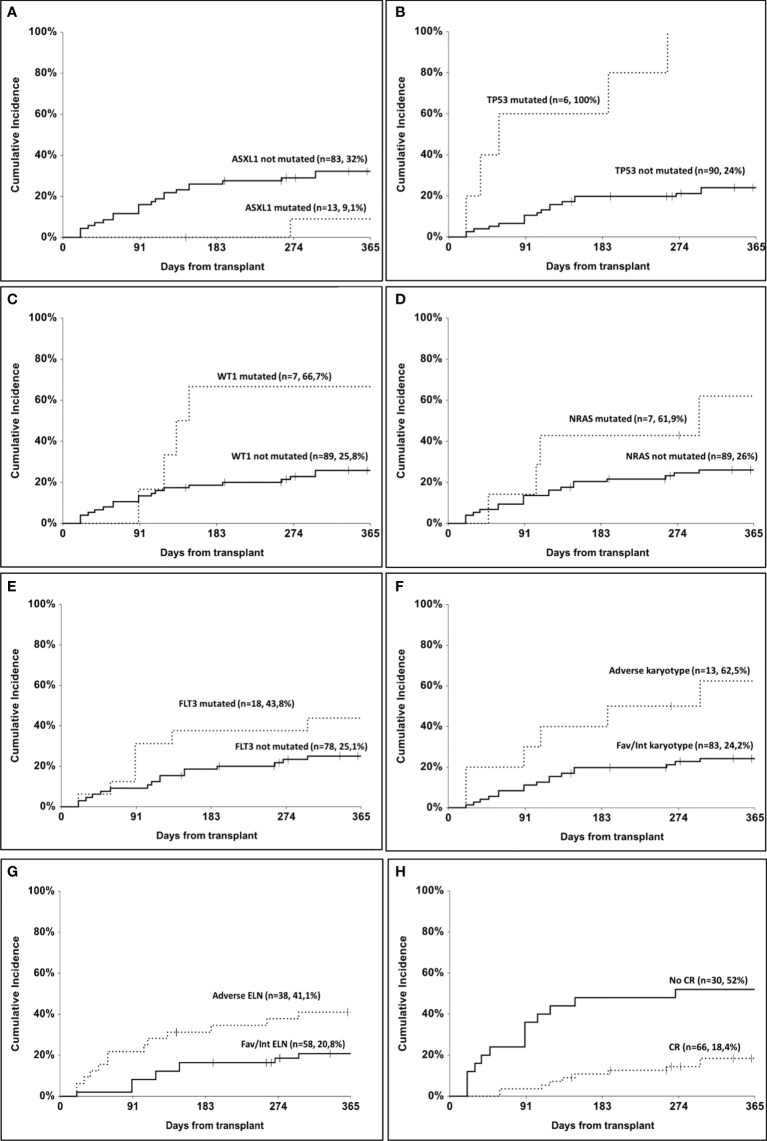
One-year cumulative incidence of relapse. **(A)** according to ASXL1 mutation; **(B)** according to TP53 mutation; **(C)** according to WT1 mutation; **(D)** according to NRAS mutation; **(E)** according to FLT3 mutation; **(F)** according to cytogenetic risk; **(G)** according to ELN risk; **(H)** according to remission status at transplant.

The overall relapse rate in our cohort was 27% (n=26). One-year cumulative incidence of relapse according to ASXL1 mutation was 32% (95% CI, 22.8–45.5) in the non-mutated and 9.1% (95% CI, 1.4–58.9) in the mutated group (p=0.05, **Figure 1A**). According to TP53 mutational status, 1-year cumulative incidence of relapse was 24% (95% CI, 16.1–36) in the non-mutated group and 100% in the mutated one (p<0.001, **Figure 1B**). In patients mutated for WT1, 1-year cumulative incidence of relapse was 66.7% (95% CI, 37.9–100) as compared to 25.8% (95% CI, 17.5–38) in non-mutated patients (p=0.04, **Figure 1C**). Patients carrying NRAS mutation showed a 1-year cumulative incidence of relapse of 61.9% (95% CI, 32.9–100) as compared to 26% (95% CI, 17.7–38.3) in the non-mutated group (p=0.05, **Figure 1D**). A trend was observed according to FLT3 mutation, with a 1-year cumulative incidence of relapse of 25.1% (95% CI, 16.4–38.4) in non-mutated patients and 43.8% (95% CI, 25.1–76.3) in mutated ones (p=0.09, **Figure 1E**). According to cytogenetic risk, 1-year cumulative incidence of relapse was 62.5% (95% CI, 37.7–100) in the adverse risk group and 24.2% (95% CI, 16–36.7) in the others (p=0.003, **Figure 1F**). According to ELN risk, 1-year cumulative incidence of relapse was 41.1% (95% CI, 37–62.4) in the adverse group and 20.8% (95% CI, 12–36.1) in the others (p=0.04, **Figure 1G**). Considering the response status at transplant, 1-year cumulative incidence of relapse was 18.4% (95% CI, 10.5–32.3) in patients who had obtained a complete remission as compared to 52% (95% CI, 35.7–75.8) in the others (p=0.004, **Figure 1H**). Finally, patients triple mutated for NPM1, FLT3, and DNMT3A (n=10) had a 1-year cumulative incidence of relapse of 50% as compared to 25.9% in others, but the difference did not reach statistical significance (p=0.07).

No statistical association was found between relapse and the other variables.

Multivariate analysis realized with Cox regression model (**Table 3**) confirmed complete remission before transplant (HR, 0.23; p=0.001), donor age (HR, 0.97; p=0.04), and adverse cytogenetic risk (HR, 3.03; p=0.04) together with FLT3 mutation (HR 3.36, p=0.02), NRAS mutation (HR 4.78, p=0.01), TP53 mutation (HR 4.34, p=0.03) and WT1 mutation (HR, 5.97; p=0.005) as independent variables for relapse occurrence.

**Table 3:** Cox regression for disease-free survival.

	Univariate			Multivariate		
Variable	HR	95% CI	p	HR	95% CI	p
TP53 mutated *vs*. not mutated	6.50	2.39–17.68	0.0002	4.34	1.11–16.99	0.03
WT1 mutated *vs*. not mutated	3.06	1.01–8.93	0.04	5.97	1.70–20.99	0.005
NRAS mutated *vs*. not mutated	2.75	0.94–8.03	0.06	4.78	1.34–17.02	0.01
Cytogenetic risk adverse *vs*. others	3.55	1.47–8.55	0.005	3.03	1.12–9.02	0.04
FLT3 mutated *vs*. not mutated	2.05	0.89–4.72	0.09	3.36	1.19–9.54	0.02
ELN risk adverse *vs*. others	2.19	1.01–4.76	0.04	1.95	0.77–4.96	0.1
Donor age continuous	0.96	0.93–0.99	0.006	0.97	0.93–0.99	0.04
Complete remission at SCT yes *vs*. no	0.34	0.16–0.74	0.007	0.23	0.09–0.56	0.001

### Overall survival and TRM

One-year OS of our study cohort was 71.5%. According to mutational status, only TP53 mutation appeared to negatively affect OS (50% *vs*. 71.7%, p=0.009). WT1 mutation made worse 1-year OS (43% *vs*. 74%, p=0.09), and ASLX1 mutation ameliorated 1-year OS (92% *vs*. 67%, p=0.06), but statistical significance was not reached in these cases. The Cox regression model (**Table 3**) identified other variables as associated with OS in univariate analysis (**Table 4**). However, multivariate analysis confirmed only relapse occurrence (HR, 4.33; p=0.0001) and grade 2–4 acute GvHD (HR, 2.29; p=0.05) as independent variables for mortality.

**Table 4:** Cox regression for overall survival.

	Univariate			Multivariate		
Variable	HR	95% CI	p	HR	95% CI	p
Patients age continuous	1.03	0.99–1.06	0.05	0.99	0.96–1.03	0.8
Lines of previous therapy continuous	1.64	0.99–2.71	0.05	1.19	0.65–2.20	0.6
HCT-comorbidity index continuous	1.26	1.06–1.51	0.009	1.13	0.94–1.36	0.2
Cytogenetic risk adverse *vs*. others	3.04	1.42–6.53	0.004	1.46	0.61–3.50	0.4
Complete remission at SCT yes *vs*. no	0.47	0.25–0.89	0.02	0.58	0.27–1.23	0.1
Conditioning reduced intensity *vs*. myeloablative	2.25	1.13–4.48	0.02	1.67	0.70–4.00	0.2
Acute GvHD 2–4 yes *vs*. no	3.31	1.66–6.59	0.0007	2.29	1.00–5.31	0.05
Relapse yes *vs*. no	6.21	3.19–12.11	<0.0001	4.33	2.07–9.06	0.0001
TP53 mutated *vs*. not mutated	3.34	1.28–8.70	0.01	1.16	0.41–3.32	0.8

No association was found between mortality and the other variables.

Regarding TRM, 1-year cumulative incidence according to TP53 mutation was 17% in non-mutated and 67% in mutated patients (p=0.002). However, multivariate Cox regression analysis confirmed only grade 2–4 acute GvHD as an independent variable for TRM (HR, 3.43; p=0.02).

No association was found between TRM and the other variables.

## Discussion

Allogeneic hematopoietic stem cell transplantation represents a potentially curative option for AML. Undoubtedly, patients and donor characteristics and transplant type might affect post-transplant outcome and TRM. On the other hand, molecular and cytogenetic features of the underlying disease surely affect the post-transplant relapse rate. The next-generation sequencing represents a valuable tool for molecular sequencing in AML diagnostic process, boasting a high sensitivity as compared to other molecular laboratory techniques. Given the growing number of genes required for diagnostic and prognostic classification, conventional approaches may be insufficient for the current stratification of AML patients (29). However, as there is still no definition of universal standard quality criteria for NGS, its application to routine diagnostic laboratories must be individually validated (7). Here, we studied the impact of the molecular mutational status of AML on relapse occurrence after HSCT.

We found four molecular mutations to be predictive of relapse occurrence 1 year after HSCT.

TP53 mutations conferred a four-times high risk for relapse (100% *vs*. 24%) as compared to wild-type patients, and increased three times the risk of death, even if this was not confirmed in multivariate analysis. It is well known that mutated TP53 is one of the main escape mechanisms adopted by neoplastic cells (30), and it is detected in <10% of *de novo* AML cases (31). Patients with TP53 mutations have a very poor prognosis, even when submitted to HSCT in CR (32, 33). In our study, 1-year cumulative incidence of relapse was 24% in the non-mutated and 100% in the mutated group (p<0.001).

The second mutated gene that increased approximately three times the risk of relapse after HSCT was FLT3. A few years ago, the Leukemia Working Party of the European Group of Blood and Bone Marrow Transplantation published data on 702 patients with normal karyotype AML who had received HSCT in CR1. The presence of FLT3 mutations, rather than NPM1, increased twice the risk of relapse and made worse overall survival but did not affect NRM (34). In our experience, we observed a 1-year cumulative incidence of relapse of 25.1% in non-mutated patients and 43.8% in mutated ones (p=0.09). Zhang et al. studied the NGS molecular profile of 332 AML patients submitted to HSCT. Multivariate analysis revealed FLT3 high allelic ratio and TP53 mutations together with MRD positivity, the lack of a CR1 before transplant, and intermediate or adverse cytogenetic risk as predictive variables for relapse occurrence (35).

The third mutated gene that heavily (~six times) affects the relapse occurrence after HSCT is WT1, which additionally worsens overall survival, although it did not reach statistical significance. In our cohort of patients mutated for WT1, 1-year cumulative incidence of relapse was 66.7% as compared to 25.8% in non-mutated patients (p=0.04). Recently, Eisfeld reported how WT1 mutation might refine ELN risk assessment in *de novo* AML. WT1 mutation among non-core-binding factor AML worsened CR rate, OS, and DFS in patients belonging to the favorable-risk group, as they were in the intermediate one. In the same way, WT1 mutation among patients in the intermediate risk group conferred CR rate, DFS, and OS similar to those registered in the high-risk group. Moreover, if isolated WT1 mutation get a worse CR rate as compared to non-mutation, the combination with mutated NPM1 negatively affected DFS and OS even when compared to sole WT1-mutated patients (36).

Quek and colleagues studied the AML mutation profile associated with disease relapse after HSCT. They registered an increased risk of relapse for patients carrying TP53, WT1, DNMT3A, and FLT3 mutations, although only WT1 and DNMT3A were confirmed in multivariate analysis. TP53 mutation also resulted in a worse relapse-free survival and overall survival, while IDH1 mutation appeared to reduce relapse occurrence and improve survival after HSCT (32). In another study by Kuskin et al., the mutational profile study of AML submitted to HSCT confirmed an increased risk of relapse in patients with FLT3, TP53, and WT1 mutations, while a lower risk of relapse was found for isolated DNMT3A-mutated patients. When they performed a subgroup analysis on patients transplanted in CR, only TP53 maintained its prognostic effect on relapse, and FLT3 showed only a trend in this sense (33).

In our cohort, NRAS is the last mutated gene that increased approximately five times the risk of relapse occurrence after HSCT. NRAS is a proto-oncogene that can be found mutated in 12% of AML cases (31). The only data we found in the literature about the role of NRAS in AML relapse regarded the identification of this mutation in approximatively 12% of patients with AML relapsing after HSCT (37). NRAS has been reported as one of those genes responsible for chemotherapy or hypomethylating treatment failure and increased mortality rate (38, 39). Fleming and colleagues recently presented the results of a machine learning model of AML risk classification according to karyotype and molecular mutations, which was conducted in a large cohort of patients (n=1,961). Isolated mutation of NRAS belonged to the poor risk group, conferring a 4-year OS of 31% that can reach 50% after HSCT in first complete remission (1CR). The association of NRAS with CEBPA or NPM1 and cohesion mutations belonged to the very good risk group, with a 4-year OS of 96%, which fell down to 80% after HSCT (40).

In contrast to data reported by the ELN risk stratification (7), in our population, ASXL1 mutation showed a protective role against relapse (9% *vs*. 32%) and ameliorated survival (92% *vs*. 67%), but these data were not confirmed by the Cox regression model. This was most probably because none of our patients with ASXL1 mutation had adverse karyotype. In our opinion, HSCT may overcome the deleterious effect of this mutation. A similar result for ASXL1 was published by Grimm and colleagues (41). They also confirmed the prognostic role of ELN classification on the prediction of OS and relapse occurrence. Moreover, they reported worse OS and relapse rates for patients with minimal residual disease (MRD) positivity at transplant and those patients with TP53 mutation, either in the setting of adverse karyotype (41). Another observation that we made regarded patients with triple positivity for NPM1, DNMT3A, and FLT3, occurring in 10 patients, who experienced a double risk of relapse after HSCT (50% *vs*. 26%), although a statistical significance was not reached. In other studies, AML patients with concurrent mutated NPM1, DNMT3A, and FLT3 genes showed poor overall survival (29, 42), but we did not observe it. Again, HSCT may have a relieving role in this association.

Finally, relapse recurrence in our cohort of patients was driven by the adverse cytogenetic risk category as compared to favorable/intermediate ones (62% *vs*. 24%) with a three times high risk, whereas the adverse ELN risk category appeared to promote relapse only in univariate analysis (41% *vs*. 21%), but the data were not confirmed in the multivariate one.

It has been previously reported that AML relapse occurrence after transplant is linked to an adverse ELN genetic risk as compared to the favorable one (43). Moreover, the lack of a 1CR status before conditioning increases the risk of relapse after HSCT. It has also been described that both these two features negatively affected overall survival after HSCT (44). In our study, we confirmed a threefold high risk of relapse in patients with adverse cytogenetic risk. Concerning the AML response status before transplant, we found a significant difference in terms of relapse between complete remission and lack of complete remission, but no differences were seen between the first and second complete remission in predicting relapse after HSCT. A large multicenter study of the Center for International Blood and Marrow Transplantation (CIBMTR) evaluated the prognostic role of the ELN risk classification in predicting the post-transplant outcome of patients with AML (45). Patients in the adverse risk group reported the highest cumulative incidence of relapse (37%) and the worse overall survival (54%) and disease-free survival (45%) at 2 years. On the other hand, they found no associations between ELN risk stratification and TRM (43, 45), which we also saw in our cohort of patients.

## Conclusions

In this study, we confirm the role of mutations of WT1, FLT3, and TP53 genes as negative on the outcome of HSCT in AML patients.

However, even if the negative prognostic impact of WT1, FLT3, and TP53 on relapse is well known, we found the discovery of NRAS mutations as a new prognostic factor in that setting interesting. The main limitations of our study were its small sample size, retrospective nature, and the lack of NGS analysis performed at the relapse time; therefore, these results require prospective validation in larger cohorts of patients.

The growing knowledge of the genetic landscape of AML allows the development of new target strategies aimed at specific subgroups of patients. Also considering the scarce benefit of allogeneic stem cell transplantation in the presence of some mutations, target drugs could be proven to eradicate MRD and possibly replace the transplantation strategy in some cases or follow it as maintenance therapy. Therefore, there is a need for new clinical studies to test the use of target drugs or the combination of multiple agents as an alternative to transplantation in the adverse categories.

## Data availability statement

The data analyzed in this study is subject to the following licenses/restrictions: available by e-mail request to PC. Requests to access these datasets should be directed to PC, patrizia.chiusolo@unicatt.it.

## Ethics statement

The studies involving human participants were reviewed and approved by Ethic committee of the Fondazione Policlinico Universitario Agostino Gemelli IRCCS in Rome (Protocol ID 4417). The patients/participants provided their written informed consent to participate in this study.

## Author contributions

EM, PC, and VA contributed to conception and design of the study. MR, GM and VA organized the database. EM, SB and SG performed the statistical analysis. EM, FF and ML wrote the first draft of the manuscript. LP, AB and SS wrote sections of the manuscript. All authors contributed to manuscript revision, read, and approved the submitted version.
